# Aphorisms in Fracture

**Published:** 1878-03

**Authors:** Richard O. Cowling

**Affiliations:** Professor of Surgical Pathology and Operative Surgery in the University of Louisville


### Miscellaneous.

### Aphorisms in Fracture.

A Paper read before the Kentucky Central Medical Association at its meeting in Harrodsburg, Kentucky, July, 1877. by RICHARD O. COWLING, A. M., M. D., Professor of Surgical Pathology and Operative Surgery in the University of Louisville.

*Gentlemen of the Kentucky Central Medical Association:*

I must offer an explanation for some peculiarities which will be found to exist in the paper prepared by me to be read upon this occasion. It was the suggestion of your president that I should write upon fractures, a suggestion which I gladly accepted, as it relieved me from the necessity of choosing a subject (which is half the battle), and at the same time it offered a most interesting theme for consideration. It was easy to see, however, that the subject was one so vast that I must either subdivide it or give but a very cursory glance at it as a whole. I did not think that either of these plans would be satisfactory; and yet there was another method, which promised to be comprehensive, and of interest to us all. It was this: rejecting all matters of history, discussion of methods, etc., to take the prominent points which arise in fractures, concerning their diagnosis, prognosis, and treatment, and to lay them down in a series of *aphorisms.* In such a manner we can cover a vast field in comparitively a short space of time. It is, too, a very useful way of imparting and receiving information, It is of course in its very nature dogmatic, as it offers no proof for its assertions; but it is presupposed on such a subject as this that the evidence on all sides has been more or less weighed, and we can judge how far the propositions laid down accord with our several sonvictions. It is, too, a very natural way of imparting intelligence. Aphorisms form the method we use continually in conversation, where we first of all state our propositions positively, and discuss them afterward if there be any necessity. I shall be glad indeed if any members of the society will do me the honor to assent to or dispute any proposition I may make, that the truth may better be arrived at.

I have felt it necessary to apologize in this manner for the aphorisms I offer, as several of them I know very general opinion will controvert.

But one word first in regard to the subject I have taken, and I proceed with my subject. Fractures form perhaps the widest chapter in surgery of any importance, and the experience gathered concerning them is correspondingly great. There are few members of the profession who have not treated a number of cases of broken bones, and yet with all these opportunities for observation a singular difference of opinion exists on some very fundamental points. John Erichsen, the apostle of plastic dressings in England, with the force of his great name and reasoning, had six years ago converted but the single hospital of which he was a surgeon to his teachings; albeit that south of the Ohio his doctrines, with the corroborative teachings (in the main) of Gross, have such sway; and in this country the practice of the two largest medical centres, Philadelphia and New York, is at total variance. And this variance in practice also prevails through the country at large. Practitioners are very slow to change the methods taught them in their early days; both from the fact that facility in new methods is hard to acquire, and respect for ancient teachers is lasting.

It is for these reasons that one most disastrous form of dressing fracture remains to a great extent to the present time in Keutucky. I allude to the control of muscular spasm by the use of direct bandage. Such were the lessons of Dudley at Lexington and Gross at Louisville. In their accomplished hands the method was highly efficient and free from danger; but many a limb has been sacrificed to it when pursued by some of their less skillful followers.

The question of extension and counter-extension in fracture is still a vexed one. You will find in the article in Holmes by Mr. Flower that he describes a "crutch-splint" with which to exert these forces in fracture of the upper-arm, while upon the other hand the necessity for such measures is denied by a large body of surgeons even in fractures of the thigh. Men are divided upon the simple question of when to dress a fracture, and upon the apparently positive knowledge concerning the prognosis of fracture and of how to dress a fracture it would take volumes to record the literature with which surgery has been flooded. We have all felt the disadvantage of this especially in the early days of our practice;how our memory has been taxed with the multiplicity of apparatus, losing sight, in the midst of so much paraphernalia, of the fact that the principle of treating every fracture is the same.

It will be a grand day for surgery when the lumber-room which contains its fracture boxes and splints of various designs shall be destroyed. The simpler necessities of our art could easily be restored.

With such a condition of affairs as this, of course an account of fractures which attempts anything like detail must deal often with elementary points; and this must be my excuse for much that follows.

### GENERAL APHORISMS.

1. In establishing diagnosis of fracture, crepitus is the most satisfactory sign; nevertheless it is not always necessary or desirable to obtain it.

2. There is frequently a physiognomy about fractures which declares the nature of the lesion; notably in Colles' fracture of the radius, ordinary fractures of clavicle, and fractures of the femoral neck.

3. The sensation in crepitus in recent fracture is *sui generis* and unmistakable. When once detected, further attempts to elicit it should not be made, except for the benefit of an accompanying physician, who may be in doubt as to the nature of the injury.

4. Crêpitus can not be had in impacted fracture, except to the injury of the patient. It is exceedingly difficult to obtain in intracapsular fracture of the femoral neck under all circumstances.

5. "Loss of function" as a symptom is all-important in establishing diagnosis in certain fractures; notably in fracture of the cervical neck and fracture of the outer portion of the clavicle.

6. Whenever any doubt exists as to the diagnosis of fracture, the examination should be conducted under an anæsthetic.

7. The chief difficulty in the differentiation of fracture is from sprain, and not from dislocation, which has its own positive signs.

8. The prognosis in simple fracture is always favorable when the injury is confined to the shaft of the bone, even if there be comminution of the bone.

9. In mutliple fracture, when simple, the healing-process is not retarded.

10. In fracture involving a joint, more or less stiffness is always to be feared.

11. With the improved methods of treatment, the danger to life and limb in compound fracture has been reduced to such an extent that the former laws for determining the question of amputation are to be recast.

12. The best time to dress any fracture is immediately after its occurrence.

13. Temporary dressings are only to be used when the materials for permanent dressings are not to be obtained, or for the purpose of moving the patient.

14. Under proper treatment immediately instituted swelling will probably be prevented.

15. The indications for treatment of fractures are, first, reduction of the fragments of bone; second, their immobilization.

16. The dressing of all fractures is best done under an anæs thetic; which not only secures the comfort of the patient, but by its influence over muscular spasm gives the best chance for perfect reposition of the fragments.

17. Perfect immobilization is only to be obtained when the joints contiguous to the fracture are secured; and there is no law more important than this in the fractures of the lower extremity.

18. One of the commonest reasons for the failure and disaster in the treatment of fracture arises from the fact that bone and muscle only are considered, and blood-vessels and nerves are left out of sight.

19. Carved and manufactured splints generally fit nobody, and are to be rejected as not only expensive but damaging. Deal board, paste-board, and the materials for the plastic apparatus form all the appliances needed by a surgeon.

20. The application of a bandage immediately to the skin, whether as a protective or to prevent muscular spasm, has resulted in such disaster that it is one of the curiosities of surgery how it could be repeated at this day. When cotton is placed *over* such a bandage it forms an absurdity scarcely credible in a man of ordinary sense.

21. Evenness or pressure is only to be obtained by the proper lining of the splints or retaining apparatus with cotton, or in the case of compound fracture with oakum. The method of padding splints, or protecting limbs with folded lint, blanket, etc., is not only vastly inferior, but generally results in discomfort to the patient, from the close packing of the material.

22. The cotton to be used is preferably known as "batting"—"wadding" is inferior. It is of prime importance to use proper batting whenever it can be obtained. It varies greatly in quality, and only the best should be used. This is smooth, easily separated in layers, and free from foreign substances, which produce inequalities and irritation. The layers should be unbroken. It is next to impossible to make an even dressing with the broken bits which the housewife so often offers to the surgeon. It should be freely used, especially over bony prominences. A fracture in the thigh of ordinary dimensions, will generally require a "pound-roll." It is to be smoothly and evenly applied. For convenience it may be held in site while the rest of the dressing is applied by ordinary sewing-thread which does not constrict.

23. Continued extension and counter-extension are as a rule not necessary to prevent shortening in fractures. This is best done by removing the causes which lead to muscular spasm: first, by early interferencs; second, by as complete reposition of the fragments as possible; third, by the smooth application of cotton batting to the limb; fourth, by the equal pressure of the bandage going from the distal end of the limb to a point beyond the joint above the fracture; fifth, by the accurate fitting of the splints or plastic material for support; sixth, by as little interference afterwards as possible.

24. Angular deformity is best overcome by the same measures as are used in longitudinal deformity. Compresses are to be avoided as insufficient and unsurgical.

25. Bandages are made of cotton or cheap flannel (preferable for the cotton in it), with the selvage torn off, and thoroughly shrunk.

26. Plastic material consists of cotton, bandages, plaster-of-paris, eggs and flour, starch, liquid glass, etc.

27. Comfort is the sign that a fracture has been properly dressed. A certain amount of soreness may be felt after any fracture, and with some temperaments pain may be present even when the fracture is properly dressed; but the general law is that pain should speedily subside when the dressings are not at fault.

28. Frequent dressings of fractures for the purpose of examination are not only useless but hurtful.

29. Whenever it is possible, after the dressing of a fracture, it should be seen again in a few hours, and the case should receive daily attention in its earlier stages.

30. The surgeon is to regard not only the welfare of his patient, but his own reputation. To this end he ought to give fair warning as to possible ill results. As suits for malpractice have arisen oftener from fracture-cases than any other kind, it will be remembered there is one thing which the law is slow to excuse—neglect.

31. If there be a consultation between two physicians in a fracture-case, and a difference of opinion in regard to treatment arise, one should yield. Compromises in cases of this kind are apt to result badly, while fractures may do well under almost any reputable method properly pursued.

32. If in case of fracture a consultation is called of a surgeon by one who does not make special pretensions to the art, he should resign to him the conduct of the case, as on him will rest the responsibility of the out-come. In other words, he who "sets" the fracture, not he who watches it, is charged with the result.

33. Whenever a fracture occurs every physician in the community within reach is summoned. The doctor should therefore be ready to treat such cases not only for the comfort of the patient, but for his own profit.

### FRACTURES OF THE FOREARM.

34. There is but one mode of dressing necessary for all fractures of the forearm, whether these be of one bone or of both, and whatever be their situation.

*Method of Dressing.*—The pieces for dressing a fractured forearm consist, first, of cotton-batting; second, of light wooden splints; third, of bandages. The splints should extend from the elbow to the tips of the fingers; they should be a trifle wider than the wrist, to prevent lateral pressure upon the bones and the obliteration of the interosseous space; they should not be much wider, else lateral displacement may occur. For convenience they may be shaped to the arm and hand. It will always be found more convenient to envelop the arm with the cotton, instead of padding the splints with the same; and where splints are padded with the cotton it is always better to fasten this material by a few turns of ordinary sewing thread. The method of padding splints by securing the cotton with bandages interferes greatly with their plasticity and comfort.

The bones having been put in apposition by gentle extension, and the splints secured to the palmer and dorsal aspect of the arm by proper bandaging from tips of fingers to elbow, the arm is to be placed in a sling, with thumb pointing upwards, in which position the bones are half way between supination and pronation, and the interosseous space is well preserved.

The dressing, when fitted for fracture of the forearm, is not to be removed, if comfort declares that it is properly doing its work, for a week or ten days, when the splints are to be shortened, so that they shall not reach beyond the roots of the fingers—and these are to be exercised frequently to prevent stiffness.

The interosseous pad, formerly considered necessary to preserve the interosseous space, is very nearly obsolete, and should be entirely so.

35. The Pistol-splint does nothing toward preserving the interosseous space.

36. The complicated dressings for Colles' fracture of the radius are not called for, and such dressings as include a compress to correct deformity are to be condemned as unsurgical, not only at the wrist, but any where.

37. In Colles' fracture, after union has taken place, there frequently remains some of its characteristic deformity. In the young thisgenerally disappears under the play of the muscles, or sometimes in bone recently united, it may be remedied by actual compression.

38. A common result in Colles' fracture, and in fractures near the wrist in adults, and especially in the aged, is a severe and persistent neuralgia. It is best treated by the hot-water douche.

39. In fractures of both bones of the arm, and frequently after fracture of one bone, after union there is a bowing of the forearm always toward the ulnar side. Sometimes this is chiefly apparent, often real. It will frequently disappear, even when excessive, under the play of the muscles, especially in the young.

40. Stiffness of the tendons and of the wrist-joint are not confined to Colles' fracture, but may occur with other fractures of the arm. Massage and passive motion will generally effect relief.

41. Many physicians do not have clear ideas concerning the fitting of a sling. It should always be worn so that the straight side comes to the hand, and the angle to the elbow.

42. Fractures of the bones of the hand are best treated like fractures of the arm.

### FRACTURES NEAR THE ELBOW.

43. Every injury, save fractured olecranon, near the elbow-joint, whether it be fracture or dislocation, should be dressed with rectangular splints.

*Method of Dressing.*—The rectangular splints for the elbow are best made from pasteboard. The limb from the fingers to the shoulder is to be enveloped in cotton; the splints, moistened in in water, are to be applied latterally, molded and confined with bandage from hand to shoulder. At the end of a week or ten days they are to be removed, and passive motion to be gently instituted, and this had best be repeated every second or third day during the progress of the treatment.

Rectangular splints are called for in injuries near the elbow, as effecting in the best manner immobilization; and, secondly, should anchylosis result from the injury they preserve the arm in the best possible shape for its future usefulness.

44. More or less stiffness of the elbow is to be expected in every fracure occurring near this joint. If the fracture be through either condyle it can not well be avoided, as passive motion in such cases, when early instituted, is liable to prevent union of the bone; but if the bone be broken above the condyles, it can frequently be prevented by careful treatment.

45. Where decided stiffness has persisted for some time after the union of fracture near the elbow, much can be done for its relief by passive motion and massage, if faithfully pursued.

46. In fracture of the olecranon where there is no separation of the fragment, owing to the fact that the fibrous expansion of the triceps is unbroken, the arm may be dressed in an angular position. In fracture with separation of the fragments of this bone, it is necessary for their apposition that the arm be dressed in an almost straight position, but the earliest possible moment should be seized to bring the arm back to an angular position, that anchylosis may not occur with the arm straight and useless.

### FRACTURES OF HUMERUS.

47. Fractures of the humerus in the lower half are best treated by rectangular splints, as in fractures of the elbow. Fractures of the upper end require shoulder-cap, the spica, etc.

*Method of Dressing Fractures in Upper End of Humerus.*—Fractures of the upper end of the humerus, including fractures of the shaft, surgical and anatomical neck, are dressed, first, by enveloping the limb from hand to shoulder with cotton; second, by bandaging from hand to upper arm; third, by fitting cap to shoulder, and over this carrying the spica bandage. The body of the patient acts as the inside splint, the hollow being filled with a folded towel, and the arm is secured to the side by additional turns of the bandage, the forearm supported in sling.

48. Stiff joint at the shoulder is not liable to occur in extra capsular fracture.

### FRACTURE OF CLAVICLE.

49. Two methods of dressing a fractured clavicle are worthy of chief consideration. These are: first, for temporary purposes, an ordinary sling, lifting forearm to an acute angle across the breast, with a band passing around the body, confining the arm closely to the side. For a permanent dressing Sayre's method, by adhesive strips, is the most convenient and efficient.

50. Whenever the clavicle is broken at its great convexity, and shows the characteristic deformity, perfect apposition is difficult to effect, or to maintain, and deformity will remain to a greater or less degree after union has taken place. It is more likely to be prevented by keeping the patient in a recumbent posture, or by using means to fix the scapula.

51. Fractures of the outer third of the clavicle are frequently overlooked by physicians, the injury being mistaken for a sprain. Displacement is not liable to occur in this situation, and crepitus is difficult to elicit. Wherever, after a fall or other injury, sharp pain is developed by pressure upon the outer third of the clavicle, fracture at this point is to be suspected.

52. The axillary pad in Fox's apparatus can not be worn with comfort, and is of very doubtful utility.

### FRACTURES OF THE RIBS.

53. In fractures of the ribs the jack-towel, or better the roller, is our chief reliance. Anhesive strips or collodion and gauze, over and around the seat of fracture, are generally not practicable, and not always efficient to conquer pain, which is the main thing we are to attack.

### FRACTURES OF THE JAW.

54. In fractures of the jaw our chief reliance is in Barton's, Gibson's, or the four-tail bandage, and soft food. Internal apparatus (inter-dental splints or tying the teeth together) practically amounts to little.

### FRACTURES OF THE NOSE.

55. In fractures of the nose the main thing is early interference, and reposition of the fragments. Apparatus to keep the bones in place afterward are principally theoretical.

### FRACTURES OF THE PELVIS.

56. In fractures of the pelvis our chief reliance is in relaxation of the abdominal muscles, and control of these from spasm from cough, etc. This is best done by the inclined plane and anodynes.

### FRACTURES OF THE LOWER EXTREMITY.

57. The proper dressing for every fracture of the lower extremity is the plastic apparatus.

*Method of making Plastic Apparatus.*—In all cases the cotton should come first; next, thread holding it in place till it can be secured evenly by bandages, and on top of this the stiffening material.

*Plaster-of-paris.*—To be fresh, finely ground (dental plaster), and well rubbed into slazy bandages of cheese lining, not more than three yards long, these to be dipped in water and applied in two or three layers over limb.

*Flour and Eggs.*—Whites to be well separated from fresh eggs, and thoroughly beaten to a froth; sifted flour to be stirred in to make a paste, which is to be rubbed into cotton bandages as they are caried over the limb, in three or four layers.

*Starch.*—Method of ,application the same, except it generally requires the addition of splints for proper stiffness.

58. The plastic apparatus in fractures of the lower extremity is not only the best of dressing, but most comfortable to patient and surgeon.

59. Failure or disaster with the plastic apparatus in fractures of the lower extremity has been due generally to its improper application, or to causes which would have operated had it not been used.

60. The chief causes of failure with the plastic apparatus have been: first, the absence of cotton as a foundation, or its scant or irregular application; second, unequal pressure of retaining bandages; third, improper material; fourth, neglect to secure the upper joint, especially a neglect of the *spica in fracture of the thigh.*

61. Under the properly applied plastic apparatus, swelling is not liable to occur.

62. In recent fracture, and above all in compound fracture, plaster-of paris is to be preferred in the manufacture of the plastic apparatus. After union has taken place, other materials may be used from consideration of lightness, etc.

63. Plaster-of-paris bandages are generally sufficiently firm for their purposes inside of thirty minutes; the flour and egg mixture within twelve hours, with manilla paper somewhat earlier; prepared chalk and gum, ozide of zinc and glue,and starch seldom harden under forty-eight to seventy hours.

64. Particular care should be taken to keep the foot at right-angles during the application of the plastic apparatus.

65. In compound fracture it is not necessary, as a general thing, to cut a trap in the apparatus under a week or a fortnight.

66. The "burning heel," which is so apt to come on soon after the application of the plastic apparatus, is best remedied by shifting the position of the limb. It seldom requires a division of the apparatus.

67. The plastic apparatus is to be cut and tied with loop bandages, when from force of circumstances the surgeon can not watch it, or for swelling or shrinking of limb, otherwise it may pass on to the end unopened.

68. While it is possible for the patient to be moved immediately after the application of the plastic apparatus to the lower extremity, and even for him to go on crutches, he is best in bed during the earlier stages of his treatment.

69. It is possible, but not probable, that fractures of the thigh may heal without shortening.

70. It is most probable that those fractures of the thigh will heal without shortening, which occur in the young, towards the lower end of the shaft, which are dressed under an anæsthetic early, by the plastic apparatus, well applied, and carried above the pelvis; nevertheless, fractures even of the upper third in stout adults *may* so heal when dressed as above described.

71. Shortening of the lower extremity under an inch can be concealed by obliquity of pelvis, or by an additional leather or so to the heel.

72. Measurement of the lower limbs from anterior spinous process to maleolus, can not be accurately done. It is better to put one end of the tape ot the umbilicus, and carry it in succession around either foot back to the point of departure. The difference indicated in this manner is twice the difference between the lengths of the limbs.

73. In fractures of the tibia or fibula abont ankle, stiffness of this joint for several months is generally inevitable. Use and massage form the treatment.

74. In fractures of the thigh high up there is tendency of bone to bow outward This may show itself for some time after the union of the bone. If not excessive it generally disappears under the play of the muscles.

75. If extension and counter-extension should be demanded in fractures of the lower extremity, there are but two methods worthy of consideration; one by "Buck's" weight and pulley, the other by Smith's anterior splint, or methods on the same principle.

76. Extension and counter-extension practiced by means of the long splint, perineal bands, etc., are useless. If the force is exerted sufficiently to have any influence on the muscles, the perineal band becomes unbearable. The apparatus speedily becomes disarranged, and requires constant professional supervision. Whatever use the long splint may have, is in correcting somewhat angular deformity. —*Louisville Med. News.*

